# Clinical spectrum and currently available treatment of type I interferonopathy Aicardi–Goutières syndrome

**DOI:** 10.1007/s12519-022-00679-2

**Published:** 2023-01-17

**Authors:** Giovanni Battista Dell’Isola, Gianluca Dini, Kaleb Logan Culpepper, Katherin Elizabeth Portwood, Pietro Ferrara, Giuseppe Di Cara, Alberto Verrotti, Mauro Lodolo

**Affiliations:** 1 Department of Pediatrics, University of Perugia, Giorgio Menghini Square, 06129 Perugia, Italy; 2 Department of Neurology Jacksonville, University of Florida, Florida, USA; 3 Department of Pediatrics, Division of Child Neurology, University of Florida, UF Health Shands Children’s Hospital, Gainesville, FL USA; 4 Unit of Pediatrics, Campus Bio-Medico University, Rome, Italy

**Keywords:** Aicardi–Goutières syndrome, Immunosuppressive drugs, Interferon-α, Neuroinflammation, Systemic lupus erythematosus

## Abstract

**Background:**

Aicardi–Goutières syndrome (AGS) is a genetically determined disorder with a variable phenotype. Since the original description of AGS, advances in gene sequencing techniques have resulted in a significant broadening of the phenotypic spectrum associated with *AGS* genes, and new clinical pictures have emerged beyond the classic presentation. The aim of this review is to provide a comprehensive analysis of the clinical spectrum of AGS and report currently available treatments and new immunosuppressive strategies.

**Data sources:**

Literature reviews and original research articles were collected from databases, including PubMed and ClinicalTrials.gov. Relevant articles about AGS were included.

**Results:**

The involvement of the nervous system certainly represents the major cause of mortality and morbidity in AGS patients. However, other clinical manifestations, such as chilblains, hepatosplenomegaly, and hematological disturbances, may lead to the diagnosis and considerably impact the prognosis and overall quality of life of these patients. Therapeutic approaches of AGS are limited to interventions aimed at specific symptoms and the management of multiple comorbidities. However, advances in understanding the pathogenesis of AGS could open new and more effective therapies.

**Conclusions:**

The over-activation of innate immunity due to upregulated interferon production plays a critical role in AGS, leading to multi-organ damage with the main involvement of the central nervous system. To date, there is no specific and effective treatment for AGS. New drugs specifically targeting the interferon pathway may bring new hope to AGS patients.

## Introduction

**Fig. 1:**
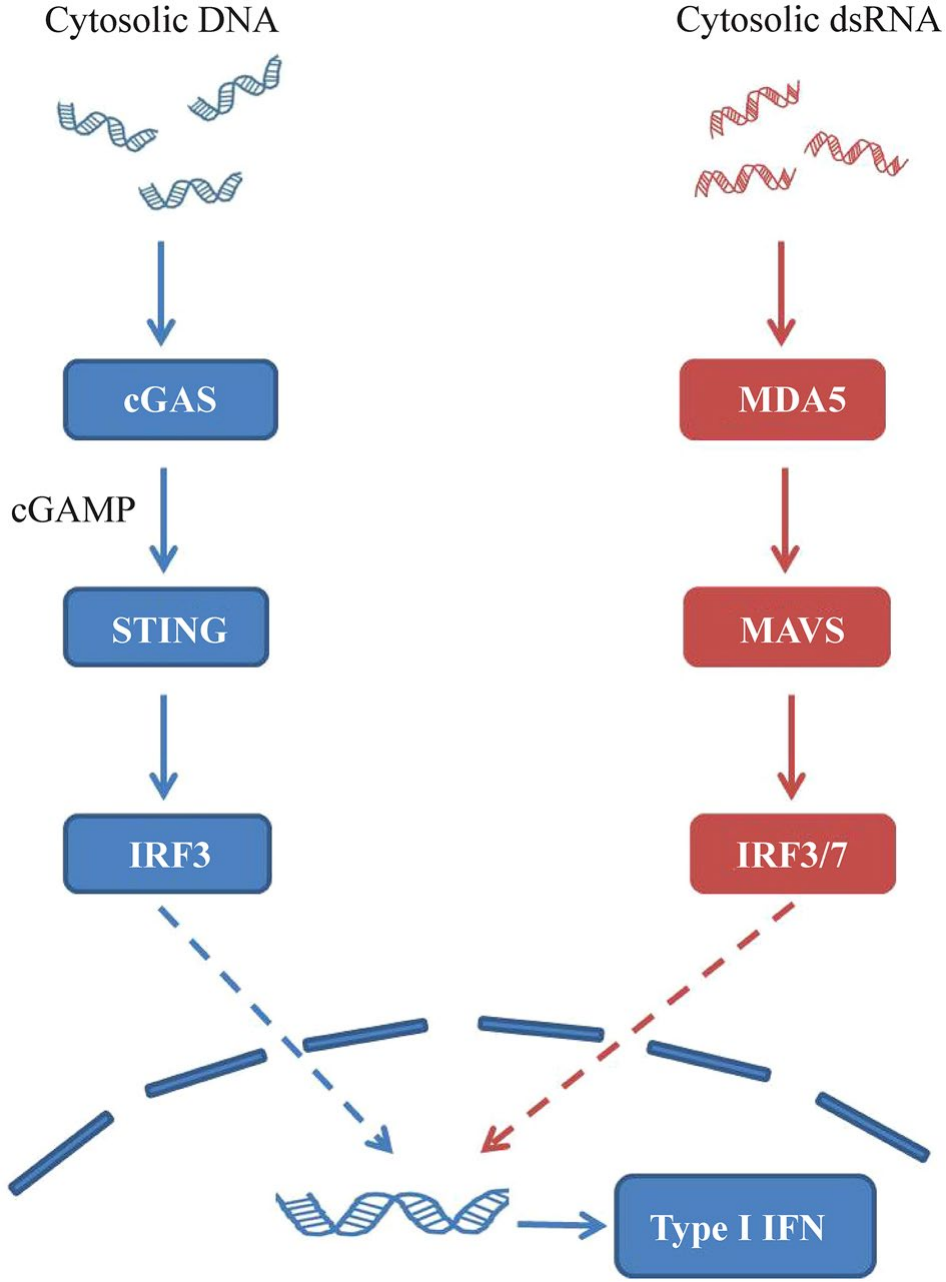
dsDNA interacts with cyclic GMP–AMP synthase (cGAS), which converts ATP and GTP to the second messenger 2′3′ cyclic GMP–AMP (cGAMP). In the endoplasmic reticulum, cGAMP binds and activates STING, leading to activation and phosphorylation of IRF3 by TANK-binding kinase 1. IRF3 forms homodimers and trans-locates into the nucleus to induce type I IFN expression. The RNA sensing pathway is also involved in AGS as a result of activation of the *MDA5*/MAVS pathway. *STING* stimulator of interferon genes, *IRF3* interferon regulatory transcription factor 3, *IFN* interferon, *AGS* Aicardi–Goutières syndrome, *MDA5* melanoma differentiation-associated gene 5, *MAVS* mitochondrial antiviral signaling

Aicardi–Goutières syndrome (AGS) is a genetically determined encephalopathy caused by mutations in any one of the nine genes [*TREX1* (3' repair exonuclease 1), *RNASEH2A* (ribonuclease H2 subunit A), *RNASEH2B*, *RNASEH2C*, *SAMHD1* (SAM-domain- and HD-domain-containing protein 1), *ADAR1* (adenosine deaminase acting on RNA 1), *IFIH* (interferon induced with helicase C domain 1), *LSM11*, and *RNU7-1*] [1–3]. Mutations in these genes affect the sensing and/or metabolism of nucleic acids, triggering an autoimmune response with an increase in interferon-α (IFN-α) production (Fig. 1). AGS usually shows an autosomal recessive pattern of inheritance. However, autosomal dominant mutations in *ADAR1*, *TREX1* and *IFIH1* have also been described [4].

AGS patients can demonstrate heterogeneous phenotypes, and beyond the central nervous system, other organs, such as the skin, thyroid, eyes and blood vessels, can be involved with high variability. AGS often leads to severe intellectual and physical disability, although some patients with normal intelligence have been described [5]. The clinical course of AGS is highly variable. In some cases, exitus occurs in the first years of life, while in others, survival occurs beyond adolescence and adulthood [2]. Radiologically, the disease is characterized by intracranial calcification, white matter abnormalities and cerebral atrophy. These patients also have chronic cerebrospinal fluid (CSF) lymphocytosis and high levels of IFN-α and neopterin [6]. Therapeutic approaches for AGS are limited to managing symptoms. However, progress in understanding AGS pathogenesis has led to targeted treatments, even though their efficacy is still to be proven.

The aim of this review is to provide a comprehensive analysis of the clinical spectrum of AGS and report currently available treatments and new immunosuppressive strategies.

## Central nervous system involvement

Since the original description of AGS, advances in gene sequencing techniques have resulted in a significant broadening of the phenotypic spectrum associated with AGS genes, and new clinical pictures have emerged beyond the classic presentation. In general, we can define several clinical scenarios characterized by significant variability in symptoms, age of onset and disease course, with marked variation in disease expression within families and across genotypes [7].

According to the literature, two clinical phenotypes could be delineated: an early-onset neonatal form, highly reminiscent of congenital infections (“pseudo-TORCH”), and a later-onset AGS that appears to be the most heterogeneous. Indeed, *AGS* genes were recognized as being associated with phenotypes different from classic AGS, such as *ADAR1*-related bilateral striatal necrosis (BSN), hereditary spastic paraplegia, and *SAMHD1*-related cerebrovascular disease [8] (Table 1).

**Table 1:** Aicardi–Goutières syndrome phenotypes and most often associated genes

Distinguishing features	Associated genes
Neonatal-onset disease	*TREX1*
Bilateral striatal necrosis, severe dystonia	*ADAR1*
Cerebrovascular disease (intracranial stenosis and aneurysms), mouth ulcers, arthropathy and glaucoma	*SAMHD1*
Hereditary spastic paraplegia	*ADAR1*, *IFIH1*, *SAMHD1* and *RNASEH2B*

*TREX1* 3' repair exonuclease 1 gene, *ADAR1* adenosine deaminase acting on RNA 1 gene, *SAMHD1* SAM-domain- and HD-domain-containing protein 1 gene, *RNASEH2B* ribonuclease H2 subunit B gene

### Prenatal-onset Aicardi–Goutières syndrome

Neonatal presentation, with disease onset occurring in utero, represents approximately 20% of AGS cases [9]. Early-onset AGS is the most frequently associated with *TREX1* mutation, but other gene mutations are also possible. These patients have a clinical picture resembling the consequences of a congenital viral infection, characterized by severe neurological compromise. The newborn usually presents jittery behavior, sterile pyrexias and poor feeding ability. In early-onset disease, neurologic findings include spasticity with paroxysmal dystonic movements, truncal hypotonia, seizures and lack of head control. Hearing is almost invariably normal. In contrast, visual function may be impaired: cases of cortical blindness and an increased risk of congenital glaucoma have been described. Neonatal presentation is associated with significantly worse neurologic outcomes and a higher risk of death. Few of these children survive beyond childhood.

Patients with early-onset disease may also present hepatosplenomegaly with hypertransaminasemia and hematological disturbances, such as anemia and thrombocytopenia, requiring transfusion therapy in some cases. AGS is associated with increased type I IFN production. IFN exerts an inhibitory effect on the proliferation and differentiation of hematopoietic cells [10], and anemia is a well-known side effect of IFN therapy [11]. IFN-α also inhibits megakaryocyte colony growth in essential thrombocythaemia [12]. However, it remains to be clarified whether these hematological disorders are due to aberrant IFN pathway activation or to other mechanisms. Furthermore, transgenic mice overexpressing IFN-α develop progressive inflammatory encephalopathy with calcium deposits and gliosis [13]. These findings are remarkably similar to those observed in patients with AGS and support the idea of IFN-α as a key factor in the pathogenesis of the disease. This hypothesis may explain the phenotypic overlap of AGS with congenital TORCH infections that also trigger an IFN-mediated response.

Magnetic resonance imaging (MRI) in these patients usually shows intracranial calcifications, white matter abnormalities and global cerebral atrophy [14]. Differential diagnosis is particularly challenging, as the combination of calcification, cerebral atrophy and white matter abnormalities may be seen in a number of early-onset disorders, such as congenital infections (e.g., cytomegalovirus). Additionally, inherited disorders, including Alexander’s disease and other leukodystrophies, can initially mimic the abnormalities present in AGS.

### Later-onset Aicardi–Goutières syndrome

Postnatal presentation of AGS includes a heterogeneous spectrum of clinical phenotypes. In most cases, after several months of apparently normal development, the affected infant presents features of a subacute encephalopathy characterized by irritability, inconsolable crying, poor feeding and intermittent fever without recognizable infectious causes (sterile pyrexias) [15]. This subacute onset is followed by a stabilization phase, and the clinical picture becomes more stable or slowly progressive. Symptoms evolve over the course of months with a delay in psychomotor development, loss of acquired skills, a slowdown in the growth of head circumference (acquired microcephaly), limb spasticity, truncal hypotonia and poor head control [16]. *RNASEH2B* is the gene most frequently associated with this later presentation [17].

### *ADAR1*-related bilateral striatal necrosis

Mutations in *ADAR1* are associated with rare and heterogeneous conditions, encompassing severe, congenital progressive forms or mild phenotypes, with delayed onset and stable course. *ADAR1* variants were observed to cause a phenotype presenting at a few months of age or in later childhood with four-limb dystonia and radiological evidence of BSN. Disease onset can occur at any time from the neonatal period through adolescence, and symptoms may begin after a nonspecific febrile infection [18]. The affected child may show signs of extrapyramidal involvement, bradykinesia, gait disturbance and dystonic posturing. During the course of the disease, some of these patients develop cognitive impairment [19], while in others, intellectual abilities are preserved. The mechanism by which *ADAR1* mutation predisposes to BSN has not yet been elucidated. Curiously, the fronto-striatal region is compromised in patients with human immunodeficiency virus (HIV) infection [20], supporting the hypothesis of a common pathogenesis between AGS and viral infections.

### Hereditary spastic paraplegia

Mutations in *ADAR1*, *IFIH1*, *SAMHD1* and *RNASEH2B* can cause a phenotype of spastic paraplegia [21, 22] with normal neuroimaging and preserved intellect. The disease usually begins in the second year of life. After normal early motor development, affected infants begin to experience progressive spasticity of the lower limbs, frequent falls and walking difficulties. MRI of the brain and spine can be completely normal, while in other patients, nonspecific changes in white matter were found. Extensive calcifications in the deep white matter of the frontal lobes and at the white‒gray junction were also reported [22].

### *SAMHD1*-related cerebrovascular disease

Cerebral vasculopathy is a common manifestation in individuals affected by bi-allelic mutations in the *SAMHD1* gene. This vascular disease carries a high risk of intracerebral hemorrhage and stroke during early life and can manifest with both intracranial stenosis (in some cases reminiscent of moyamoya disease) and aneurysms. Patients can present a heterogeneous phenotype, including variable developmental disability, chilblain lesions, stenosis of the intracranial vessels, stroke, and glaucoma [23, 24]. The molecular mechanism by which *SAMHD1* mutations affect the integrity of cerebral vessels remains unclear. *SAMHD1* probably plays a protective role in preventing self-activation of innate immunity [25], and mutations of this gene predispose patients to cerebrovascular disease through immune etiology.

### Epilepsy in Aicardi–Goutières syndrome

Epilepsy is a common feature of AGS, which occurs in about one-third of patients [26] and is characterized by an early onset and refractory course [27]. Seizure semiology is variable. Ramantani [27], in a cohort of 12 patients with AGS and epilepsy, reported a prevalence of tonic seizures (69%), but myoclonic, secondary generalized and focal seizures were also described. Ramantani did not find a correlation between the severity of epilepsy and neuroimaging findings, while in a group of twenty-seven AGS patients [28], the presence of epilepsy was significantly associated with calcification severity, suggesting an anatomical correlate. The majority of affected infants present a marked startle reaction to sudden noise [18]. Electroencephalography in AGS may show a diffuse slowing of background activity and disruption of electrical organization, especially in patients with early-onset disease [28]. However, the electro-clinical basis of AGS-related epilepsy requires further elucidation.

### Laboratory results in Aicardi–Goutières syndrome

Characteristics of blood results in patients with AGS include neutropenia, anemia, thrombocytopenia and elevated liver enzymes. Multi-lineage cytopenias are a potential complication of AGS, not limited to the neonatal period. The analysis of CSF in AGS patients usually reveals chronic lymphocytosis with markedly elevated neopterin and increased levels of IFN-α, especially in the early stages of the disease, although not invariably [7]. CSF abnormalities are negatively correlated with age, with a peak in the first year of life and subsequent gradual reduction with growth [29]. Neopterin is a key component of the innate immune system and is produced by macrophages during viral or bacterial infection and autoimmune disorders [30]. It is a sensitive marker of neuro-inflammation [31], and increased levels of neopterin in patients with AGS are compatible with the dysimmune etiology of the disease.

### Neuroimaging features

Neuroimaging plays an important role in the diagnosis of AGS. The main neuroradiological features include intracranial calcifications, white matter abnormalities and cerebral atrophy. Brain calcifications are best detectable on computed tomography and are mainly localized in the basal ganglia, especially the putamen and globus pallidus, in the periventricular region and deep white matter, in the thalamus and in the dentate nucleus of cerebellum [32]. Leukodystrophy usually shows a symmetric distribution and appears on MRI as hyper-intense signals on T2-weighted images. These signal intensity alterations can have a frontal and temporal lobe predominance or show a diffuse involvement of white matter. Less frequently, a periventricular pattern has also been described. In severe cases, temporal lobe cysts can be recognized [33]. Deep white matter cysts are most frequently associated with *TREX1* mutation, but they can also be found in patients with other mutations [34]. Deep white matter cysts probably constitute a consequence of prenatal disease onset. Cerebral atrophy may be progressive and mainly affect periventricular white matter and sulci. In some cases, it is associated with brainstem thinning and cerebellar atrophy. Some radiological patterns appear to be linked to specific genotypes, such as BSN in cases of *ADAR1*-related AGS [5] and *RNASEH2B*-associated porencephalic cysts [35].

## Auto-inflammatory skin manifestation

Cutaneous findings are the most prominent extra-neurological features of AGS. More than 40% of AGS patients present skin manifestations [2]. The cutaneous findings are most often localized to the extremities and are worsened by exposure to cold. The most typical skin manifestations related to AGS are chilblain-like lesions. Chilblains can be seen in association with mutations in any of the *AGS* genes [36] and typically present as intermittent puffy swelling and necrotic areas on the hands, feet, ears and elbows [37]. Biopsy of these lesions is characterized by basal vacuolar degeneration, interface dermatitis and dermal infiltrate [37]. Beyond chilblain-like lesions, other skin manifestations in AGS include acrocyanosis, nail abnormalities, mouth ulcers and Raynaud’s phenomenon [38–41]. Mouth ulcers are most frequently found in patients carrying *SAMHD1* mutations. Mutations in AGS-associated genes have also been reported in other dermatological disorders. One case of psoriasis was described in a patient with AGS due to *IFIH1* mutation [42]. Moreover, psoriasis is reported among the effects of IFN-α therapy [43]. Recently, a case of angiokeratoma of Mibelli was described in a patient with AGS due to *RNASEHB2* mutation [44]. Acral lentiginosis has been observed in a family with *IFIH1*-related AGS [37]. *TREX1* and *SAMHD1* mutations have been associated with cases of familial chilblain lupus [45, 46].

Mutations of *ADAR1* also cause dyschromatosis symmetrica hereditaria, a rare skin condition characterized by a mixture of hyper-pigmented and hypo-pigmented macules distributed on the dorsal hands and feet [47]. In conclusion, skin lesions represent a main feature of AGS and may lead to the diagnosis, especially of later-onset forms with mild neurological involvement. Therefore, clinicians should be aware of the differential diagnosis of these signs for an early diagnosis of AGS.

## Endocrinopathies

Endocrine involvement represents one of the possible autoimmune complications of AGS. The most frequent endocrinopathies described in AGS are hypothyroidism and diabetes insipidus (DI). However, there have been reports of patients with diabetes mellitus, hyperparathyroidism, growth hormone deficiency and adrenal insufficiency [11]. Hypothyroidism and DI associated with AGS usually have a transitory course. Hypothyroidism has been documented mainly in patients carrying *TREX1* mutations, and it is generally a subclinical hypothyroidism [48]. The pathogenesis of hypothyroidism in AGS is unclear. Anti-thyro-peroxidase antibodies have been found in a minority of these patients; therefore, thyroid dysfunction may be due to a direct toxic effect of IFN [48]. Worth et al. reported two patients with AGS and DI. They both presented hypernatremia and polyuria with reduced urinary osmolarity and responded to the administration of desmopressin [49]. Although endocrine involvement is not a hallmark of AGS, periodic monitoring of thyroid function and serum electrolytes is required in these patients.

## Treatment of Aicardi–Goutières syndrome

Despite adequate treatment and follow-up, AGS severely impacts the quality of life of patients and caregivers. Hence, there is a need for new therapies aimed at preventing the evolution of the disease. Current treatments include interventions aimed at specific symptoms, such as respiratory or nutritional support. Endocrine manifestations are generally transient but may require treatment with desmopressin for DI or with levothyroxine for hypothyroidism [48]. AGS patients may present hematological disorders such as thrombocytopenia, which may require platelet transfusion. Empirical therapy with conventional immunosuppressant drugs (corticosteroids, intravenous immunoglobulin) did not show clear evidence of benefits [50]. New therapeutic strategies directed toward reducing type I INF production and/or blocking type I INF-induced signaling have been hypothesized [51]. Evaluation of treatment efficacy and neurological improvement in AGS is challenging, especially due to the variable course of the disease and the severe neurological impairment of many of the affected patients. Reduced expression of IFN-stimulated genes (IFN signature) is used as an indicator of therapeutic response [52]. New drugs currently include Janus kinase (JAK) inhibitors, such as baricitinib and ruxolitinib, and reverse transcriptase inhibitors (RTIs) (Table 2). JAK signal transducers and activators of the transcription pathway are essential for the biological activity of a wide range of cytokines. Thus, inhibition of JAK signaling represents a therapeutic goal for the treatment of various autoimmune disorders [59]. Kothur et al. reported treatment results from a patient with AGS due to *IFIH1* mutation: at 16 months of life, therapy with intravenous immunoglobulin and corticosteroids was started, but the child showed a mild clinical response. At the age of 32 months, oral ruxolitinib was administered at a starting dosage of 2.5 mg twice daily and increased to 5 mg twice daily after six weeks. The patient achieved a clinical improvement with a reduction in dystonic movements, recovery of neuro-motor skills and a positive effect on blood and CSF pro-inflammatory biomarkers [56].

**Table 2:** Main information from literature about treated Aicardi–Goutières syndrome patients

References	Genotype (number of patients)	Drug(s) used and administration regimen	Reported outcomes
Cattalini et al., 2021 [53]	*ADAR1* (1)	Ruxolitinib: 2.5–5 mg twice daily	Mild improvement of the neurological picture (reduction of bradykinesia, better fine motor skills and balance competencies, vocabulary expansion)
Vanderver et al., 2020 [54]	*TREX1* (5), *RNASEH2B* (8), *RNASEH2B* (1), *RNASEH2B* (1), *SAMHD1* (5), *ADAR1* (7), *IFIH1* (8)	Baricitinib: 0.1–0.6 mg/kg in 2 to 4 doses for a minimum of 12 mon	Reduced skin inflammation. Gain of new milestones during treatment
Meesilpavikkai et al., 2019 [55]	*SAMHD1* (1)	Baricitinib: 2 mg/kg	Complete resolution of chilblains after 6 wk of treatment
Kothur et al., 2018 [56]	*IFIH1* (1)	Oral prednisolone: 2 mg/kg and IVIG 1 g/kg Ruxolitinib: 2.5–5 mg twice daily	Temporary benefits in head and trunk control and decreased irritability and pyrexias Improved functional motor scores, lowering of blood and CSF proinflammatory biomarkers
Rice et al., 2018 [57]	*TREX1* (2), *RNASEH2B* (3), *RNASEH2A* (1), *SAMHD1* (2)	Abacavir, lamivudine and zidovudine for 12 mon	Reduced expression of type I IFN regulated genes (IFN signature)
Tüngler et al., 2016 [58]	*RNASEH2B* (2)	Ruxolitinib: 0.2–0.5 mg/kg/d	Reduced expression of type I IFN regulated genes in both patients Reduction of dystonic movements in one patient
Orcesi et al., 2008 [50]	*RNASEH2B* (1)	High-dose steroid and IVIG	No neurological improvement
D’Arrigo et al., 2008 [15]	*RNASEH2B* (2)	Methylprednisolone intravenous bolus followed by oral prednisone Subsequently IVIG: 0.4 mg/kg/d for 5 d (6 cycles at intervals of 30 d)	No neurological improvement

*IFN* interferon, *CSF* cerebrospinal fluid, *IVIG* intravenous immunoglobulin, *ADAR1* adenosine deaminase acting on RNA 1 gene, *TREX1* 3' repair exonuclease 1 gene, *RNASEH2B* ribonuclease H2 subunit B gene, *SAMHD1* SAM-domain- and HD-domain-containing protein 1 gene, *IFIH1* interferon induced with helicase C domain 1 gene

Recently, Cattalini et al. reported the use of ruxolitinib in a 5-year-old girl affected by AGS due to *ADAR1* mutation. A significant improvement in neuro-motor skills was described after 18 months of follow-up [53]. In an open-label study, 35 patients with genetically confirmed AGS received baricitinib at a dose ranging from 0.1 to 0.6 mg/kg for a minimum of 12 months. The majority of patients met new developmental milestones during the treatment period, and 12 patients gained two to seven new skills [54]. Baricitinib administration was highly effective for treating pericardial effusion in a patient with AGS [60]. Baricitinib also demonstrated a positive effect on chilblains associated with AGS [55]. However, the risks and benefits of treatment with baricitinib should be carefully considered. The primary risks associated with baricitinib among patients with AGS were thrombocytosis, leukopenia, and infection [54]. Although JAK inhibitors have shown encouraging results in some reports, they cannot act on those alterations that already occur in utero in many patients with AGS.

RTIs are widely used for the treatment of HIV infection [61]. Their use in AGS is justified by the hypothesis that RTIs can inhibit the reverse transcription of endogenous retro-elements arising from the integration of retroviruses into the human genome. The chronic detection of these retro-elements can override tolerance mechanisms for constitutive self-antigens, leading to autoimmune responses with consequent tissue damage. Thus, RTIs represent a potential therapy for AGS and other autoimmune diseases [62]. In an open-label study, 11 AGS patients were administered a combination therapy comprising three nucleoside analog RTIs (zidovudine, lamivudine and abacavir) for a treatment period of 12 months. Eight of 11 patients who were recruited completed the study. Treatment with RTIs resulted in a reduction in IFN-α levels in serum and an increase in cerebral blood flow during the period of therapy [57]. Given the role of type I IFN in the pathogenesis of AGS, blocking IFN-α signaling might represent another possible therapeutic strategy. IFN-α signaling can be blocked either with anti-IFN-α antibodies, such as sifalimumab, or anti-type I IFN receptor antibodies (anifrolumab).

To date, there are no reports or ongoing clinical trials on the use of these molecules in patients with AGS. However, their use has been reported in other diseases that recognize dysregulation of autoimmunity as a pathogenetic mechanism. For example, monoclonal antibodies directed against the anti-IFNα receptor ameliorated disease in mouse models of lupus [63]. Sifalimumab was well tolerated in patients with systemic lupus erythematosus (SLE) [64], and its efficacy was evaluated in a phase IIb study in patients with moderate to severe SLE [65]. Moreover, a clinical trial conducted on 362 SLE patients demonstrated the efficacy of anifrolumab against placebo in the control of skin and joint lesions [66]. Another theoretical strategy may be the inhibition of the cGAS-STING [cyclic GMP–AMP synthase stimulator of interferon (*IFN*) gene] pathway. Chronic activation of this pathway is implicated in the pathogenesis of AGS. Furthermore, the silencing of *cGAS* can rescue the lethal phenotype in *TREX1*^−/−^ mice [67], suggesting that cGAS inhibitors may be useful therapeutics for AGS and related autoimmune diseases. A number of molecules have been generated in attempts to inhibit the cGAS-STING pathway. These include small molecule inhibitors [68], suppressive oligo-deoxy-nucleotides [69], suramin (acting by displacing the bound DNA from cGAS) [70] and acetylsalicylic acid (can directly acetylate cGAS and efficiently suppress its activity) [71].

Finally, another therapeutic strategy previously reported is the inhibition of interleukin-6 (IL-6). Although the relationship between IL-6 production and IFN-α signaling in the context of *SAMHD1* mutation-mediated cerebral vasculopathy remains unclear [72], Henrickson et al. [73] observed a favorable response to the IL-6 inhibitor tocilizumab in a patient with homozygous *SAMHD1* mutation.

## Conclusions

AGS is a genetic encephalopathy that usually, but not always, results in severe intellectual and physical disability. There are certain cases where a classic, well-known phenotype is not present. Nevertheless, knowing all the characteristics as well as infrequent features of AGS can contribute to an early diagnosis. CNS involvement usually represents the major cause of mortality and morbidity in AGS patients. However, several organs are affected with considerable impact on the prognosis and overall quality of life of patients and their caregivers. To date, AGS therapy represents a great challenge due to the scarce availability of currently effective therapies and to the severity of the clinical picture that many patients already present at birth. The increasing knowledge about the disease mechanisms in AGS is now opening the way to novel potential treatments targeting molecular events. The future objectives concern the possibility of early diagnosis and the development of therapies that can prevent those alterations that already occur in utero. Moreover, an early diagnosis relieves the patient from the large number of diagnostic procedures searching for the cause of the condition and provides families with information about possible hereditary risks. New therapeutic strategies directed toward reducing type I INF production and/or blocking type I INF-induced signaling are under development. Unfortunately, the rarity of the syndrome and the small number of studies conducted make it difficult to prove the efficacy of novel therapies. Multicenter studies are needed to evaluate the real efficacy and safety of these treatments in AGS.

## Data Availability

All data generated or analyzed during this study are included in this published article.

## References

[1] Aicardi J, Goutières F (1984). A Progressive familial encephalopathy in infancy with calcifications of the basal ganglia and chronic cerebrospinal fluid lymphocytosis. Ann Neurol.

[2] Crow YJ, Chase DS, Lowenstein Schmidt J, Szynkiewicz M, Forte GMA, Gornall HL (2015). Characterization of human disease phenotypes associated with mutations in *TREX1*, *RNASEH2A*, *RNASEH2B*, *RNASEH2C*, *SAMHD1*, *ADAR*, and *IFIH1*. Am J Med Genet Part A.

[3] Uggenti C, Lepelley A, Depp M, Badrock AP, Rodero MP, El-Daher MT (2020). cGAS-mediated induction of type I interferon due to inborn errors of histone pre-mRNA processing. Nat Genet.

[4] Rice GI, Del Toro DY, Jenkinson EM, Forte GMA, Anderson BH, Ariaudo G (2014). Gain-of-function mutations in *IFIH1* cause a spectrum of human disease phenotypes associated with upregulated type I interferon signaling. Nat Genet.

[5] McEntagart M, Kamel H, Lebon P, King MD (1998). Aicardi-Goutieres syndrome: an expanding phenotype. Neuropediatrics.

[6] Blau N, Bonafé L, Krägeloh-Mann I, Thöny B, Kierat L, Häusler M (2003). Cerebrospinal fluid pterins and folates in Aicardi-Goutières syndrome: a new phenotype. Neurology.

[7] Livingston JH, Crow YJ (2016). Neurologic phenotypes associated with mutations in *TREX1*, *RNASEH2A*, *RNASEH2B*, *RNASEH2C*, *SAMHD1*, *ADAR1*, and *IFIH1*: Aicardi-Goutières syndrome and beyond. Neuropediatrics.

[8] Crow YJ, Shetty J, Livingston JH (2020). Treatments in Aicardi-Goutières syndrome. Dev Med Child Neurol.

[9] Crow YJ. Aicardi-Goutières syndrome. 2005 Jun 29 (updated 2016 Nov 22). In: Adam MP, Everman DB, Mirzaa GM, Pagon RA, Wallace SE, Bean LJH, et al., editors. GeneReviews^®^. Seattle (WA): University of Washington, Seattle; 1993–2022. 20301648

[10] Sata M, Yano Y, Yoshiyama Y, Ide T, Kumashiro R, Suzuki H (1997). Mechanisms of thrombocytopenia induced by interferon therapy for chronic hepatitis B. J Gastroenterol.

[11] Espinosa M, Arenas MD, Aumente MD, Barril G, Buades JM, Aviles B (2007). Anemia associated with pegylated interferon-α2a and α2b therapy in hemodialysis patients. Clin Nephrol.

[12] Gugliotta L, Bagnara GP, Catani L, Gaggioli L, Guarini A, Zauli G (1989). In vivo and in vitro inhibitory effect of α-interferon on megakaryocyte colony growth in essential thrombocythaemia. Br J Hematol.

[13] Cuadrado E, Jansen MH, Anink J, De Filippis L, Vescovi AL, Watts C (2013). Chronic exposure of astrocytes to interferon-α reveals molecular changes related to Aicardi-Goutieres syndrome. Brain.

[14] Vanderver A, Prust M, Kadom N, Demarest S, Crow YJ, Helman G (2015). Early-onset Aicardi-Goutières syndrome: magnetic resonance imaging (MRI) pattern recognition. J Child Neurol.

[15] D’arrigo S, Riva D, Bulgheroni S, Chiapparini L, Lebon P, Rice G (2008). Aicardi-Goutières syndrome: description of a late onset case. Dev Med Child Neurol.

[16] Livingston JH, Lin JP, Dale RC, Gill D, Brogan P, Munnich A (2014). A type I interferon signature identifies bilateral striatal necrosis due to mutations in *ADAR1*. J Med Genet.

[17] Rice G, Patrick T, Parmar R, Taylor CF, Aeby A, Aicardi J (2007). Clinical and molecular phenotype of Aicardi-Goutières syndrome. Am J Hum Genet.

[18] Piccoli C, Bronner N, Gavazzi F, Dubbs H, De Simone M, De Giorgis V (2021). Late-onset Aicardi-Goutières syndrome: a characterization of presenting clinical features. Pediatr Neurol.

[19] Tojo K, Sekijima Y, Suzuki T, Suzuki N, Tomita Y, Yoshida K (2006). Dystonia, mental deterioration and dyschromatosis symmetrica hereditaria in a family with *ADAR1* mutation. Mov Disord.

[20] Melrose RJ, Tinaz S, Castelo JMB, Courtney MG, Stern CE (2008). Compromised fronto-striatal functioning in HIV: a fMRI investigation of semantic event sequencing. Behav Brain Res.

[21] Crow YJ, Zaki MS, Abdel-Hamid MS, Abdel-Salam G, Boespflug-Tanguy O, Cordeiro NJV (2014). Mutations in *ADAR1*, *IFIH1*, and *RNASEH2B* presenting as spastic paraplegia. Neuropediatrics.

[22] Ruaud L, Rice GI, Cabrol C, Piard J, Rodero M, van Eyk L (2018). Autosomal-dominant early-onset spastic paraparesis with brain calcification due to *IFIH1* gain-of-function. Hum Mutat.

[23] Li W, Xin B, Yan J, Wu Y, Hu B, Liu L (2015). *SAMHD1*gene mutations are associated with cerebral large-artery atherosclerosis. Biomed Res Int.

[24] Xin B, Jones S, Puffenberger EG, Hinze C, Bright A, Tan H (2011). Homozygous mutation in *SAMHD1* gene causes cerebral vasculopathy and early onset stroke. Proc Natl Acad Sci U S A.

[25] Rice GI, Bond J, Asipu A, Brunette RL, Manfield IW, Carr IM (2009). Mutations involved in Aicardi-Goutières syndrome implicate *SAMHD1* as regulator of the innate immune response. Nat Genet.

[26] Wang W, Wang W, He TY, Zou LP, Li WD, Yu ZX (2022). Analysis of clinical characteristics of children with Aicardi-Goutieres syndrome in China. World J Pediatr.

[27] Ramantani G, Maillard LG, Bast T, Husain RA, Niggemann P, Kohlhase J (2014). Epilepsy in Aicardi-Goutières syndrome. Eur J Paediatr Neurol.

[28] De Giorgis V, Varesio C, Viri M, Giordano L, La Piana R, Tonduti D, et al. The epileptology of Aicardi-Goutières syndrome: electroclinical-radiological findings. 2021;86:197–209. 10.1016/j.seizure.2020.11.019 33589296

[29] Dale RC, Brilot F, Fagan E, Earl J (2009). Cerebrospinal fluid neopterin in pediatric neurology: a marker of active central nervous system inflammation. Dev Med Child Neurol.

[30] Fuchs D, Weiss G, Reibnegger G, Wachter H (1992). The role of neopterin as a monitor of cellular immune activation in transplantation, inflammatory, infectious, and malignant diseases. Crit Rev Clin Lab Sci.

[31] Molero-Luis M, Casas-Alba D, Orellana G, Ormazabal A, Sierra C, Oliva C (2020). Cerebrospinal fluid neopterin as a biomarker of neuroinflammatory diseases. Sci Rep.

[32] Uggetti C, La Piana R, Orcesi S, Egitto MG, Crow YJ, Fazzi E (2009). Aicardi-Goutières syndrome: neuroradiologic findings and follow-up. AJNR Am J Neuroradiol.

[33] La Piana R, Uggetti C, Roncarolo F, Vanderver A, Olivieri I, Tonduti D (2016). Neuroradiologic patterns and novel imaging findings in Aicardi-Goutières syndrome. Neurology.

[34] Oleksy B, Mierzewska H, Tryfon J, Wypchło M, Wasilewska K, Zalewska-Miszkurka Z (2022). Aicardi-Goutières syndrome due to a *SAMHD1* mutation presenting with deep white matter cysts. Mol Syndromol.

[35] Abdel-Salam GMH, Abdel-Hamid MS, Mohammad SA, Abdel-Ghafar SF, Soliman DR, El-Bassyouni HT (2017). Aicardi-Goutières syndrome: unusual neuro-radiological manifestations. Metab Brain Dis.

[36] Abdel-Salam GMH, El-Kamah GY, Rice GI, El-Darouti M, Gornall H, Szynkiewicz M (2010). Chilblains as a diagnostic sign of aicardi-goutières syndrome. Neuropediatrics.

[37] Kolivras A, Aeby A, Crow YJ, Rice GI, Sass U, André J (2008). Cutaneous histopathological findings of Aicardi-Goutières syndrome, overlap with chilblain lupus. J Cutan Pathol.

[38] Juern A, Robbins A, Galbraith S, Drolet B (2010). Aicardi-Goutières syndrome: cutaneous, laboratory, and radiologic findings: a case report. Pediatr Dermatol.

[39] Singh S, Taneja N, Bala P, Verma KK, Devarajan LSJ (2018). Aicardi-Goutières syndrome: cold-induced acral blemish is not always cryoglobulinaemic vasculitis or chilblain lupus. Clin Exp Dermatol.

[40] Wu D, Fang L, Huang T, Ying S (2021). Case report: Aicardi-Goutières syndrome caused by novel *TREX1*variants. Front Pediatr.

[41] Yarbrough K, Danko C, Krol A, Zonana J, Leitenberger S (2016). The importance of chilblains as a diagnostic clue for mild Aicardi-Goutières syndrome. Am J Med Genet Part A.

[42] Zheng S, Lee PY, Wang J, Wang S, Huang Q, Huang Y (2020). Interstitial lung disease and psoriasis in a child with Aicardi-Goutières syndrome. Front Immunol.

[43] Afshar M, Martinez AD, Gallo RL, Hata TR (2013). Induction and exacerbation of psoriasis with Interferon-alpha therapy for hepatitis C: a review and analysis of 36 cases. J Eur Acad Dermatol Venereol.

[44] Cinotti E, Bertello M, Habougit C, Rongioletti F, Cambazard F, Antoine JC (2021). Aicardi-Goutières syndrome: a possible explanation of angiokeratoma of Mibelli. J Eur Acad Dermatol Venereol.

[45] Rice G, Newman WG, Dean J, Patrick T, Parmar R, Flintoff K (2007). Heterozygous mutations in *TREX1* cause familial chilblain lupus and dominant Aicardi-Goutières syndrome. Am J Hum Genet.

[46] Günther C, Meurer M, Stein A, Viehweg A, Lee-Kirsch MA (2009). Familial chilblain lupus—a monogenic form of cutaneous lupus erythematosus due to a heterozygous mutation in *TREX1*. Dermatology.

[47] Miyamura Y, Suzuki T, Kono M, Inagaki K, Ito S, Suzuki N (2003). Mutations of the RNA-specific adenosine deaminase gene (*DSRAD*) are involved in dyschromatosis symmetrica hereditaria. Am J Hum Genet.

[48] Carella C, Mazziotti G, Amato G, Braverman LE, Roti E (2004). Interferon-α-related thyroid disease: pathophysiological, epidemiological, and clinical aspects. J Clin Endocrinol Metab.

[49] Worth C, Briggs TA, Padidela R, Balmer E, Skae M (2020). Endocrinopathies in Aicardi Goutières syndrome—a descriptive case series. Clin Case Reports.

[50] Orcesi S, Pessagno A, Biancheri R, La Piana R, Mascaretti M, Rossi A (2008). Aicardi-Goutières syndrome presenting atypically as a subacute leukoencephalopathy. Eur J Paediatr Neurol.

[51] Tonduti D, Fazzi E, Badolato R, Orcesi S (2020). Novel and emerging treatments for Aicardi-Goutières syndrome. Expert Rev Clin Immunol.

[52] Adang LA, Frank DB, Gilani A, Takanohashi A, Ulrick N, Collins A (2018). Aicardi Goutières syndrome is associated with pulmonary hypertension. Mol Genet Metab.

[53] Cattalini M, Galli J, Zunica F, Ferraro RM, Carpanelli M, Orcesi S (2021). Case report: the JAK-inhibitor ruxolitinib use in Aicardi-Goutieres syndrome due to *ADAR1* mutation. Front Pediatr.

[54] Vanderver A, Adang L, Gavazzi F, McDonald K, Helman G, Frank DB (2020). Janus kinase inhibition in the Aicardi-Goutières syndrome. N Engl J Med.

[55] Meesilpavikkai K, Dik WA, Schrijver B, van Helden-Meeuwsen CG, Versnel MA, van Hagen PM (2019). Efficacy of baricitinib in the treatment of chilblains associated with Aicardi-Goutières syndrome, a type I interferonopathy. Arthritis Rheumatol.

[56] Kothur K, Bandodkar S, Chu S, Wienholt L, Johnson A, Barclay P (2018). An open-label trial of JAK 1/2 blockade in progressive *IFIH1*-associated neuroinflammation. Neurology.

[57] Rice GI, Meyzer C, Bouazza N, Hully M, Boddaert N, Semeraro M (2018). Reverse-transcriptase inhibitors in the Aicardi-Goutières syndrome. N Engl J Med.

[58] Tüngler V, König N, Günther C, Engel K, Fiehn C, Smitka M, et al. Response to: “JAK inhibition in STING-associated interferonopathy” by Crow et al. Ann Rheum Dis. 2016;75:e76. 10.1136/annrheumdis-2016-210565 27811148

[59] Furumoto Y, Gadina M (2013). The arrival of JAK inhibitors: advancing the treatment of immune and hematologic disorders. BioDrugs.

[60] Casas-Alba D, Darling A, Caballero E, Mensa-Vilaró A, Bartrons J, Antón J (2022). Efficacy of baricitinib on chronic pericardial effusion in a patient with Aicardi-Goutières syndrome. Rheumatology (Oxford).

[61] Wang Y, De Clercq E, Li G (2019). Current and emerging nonnucleoside reverse transcriptase inhibitors (NNRTIs) for HIV-1 treatment. Expert Opin Drug Metab Toxicol.

[62] Stetson DB (2012). Endogenous retroelements and autoimmune disease. Curr Opin Immunol.

[63] Baccala R, Gonzalez-Quintial R, Schreiber RD, Lawson BR, Kono DH, Theofilopoulos AN (2012). Anti-IFN-α/β receptor antibody treatment ameliorates disease in lupus-predisposed mice. J Immunol.

[64] Takeuchi T, Tanaka Y, Matsumura R, Saito K, Yoshimura M, Amano K (2020). Safety and tolerability of sifalimumab, an anti-interferon-α monoclonal antibody, in Japanese patients with systemic lupus erythematosus: a multicenter, phase 2, open-label study. Mod Rheumatol.

[65] Greth W, Robbie GJ, Brohawn P, Hultquist M, Yao B (2017). Targeting the interferon pathway with sifalimumab for the treatment of systemic lupus erythematosus. Immunotherapy.

[66] Morand EF, Furie R, Tanaka Y, Bruce IN, Askanase AD, Richez C (2020). Trial of anifrolumab in active systemic lupus erythematosus. N Engl J Med.

[67] Gray EE, Treuting PM, Woodward JJ, Stetson DB (2015). Cutting edge: cGAS is required for lethal autoimmune disease in the *Trex1*-deficient mouse model of Aicardi-Goutières syndrome. J Immunol.

[68] Wiser C, Kim B, Vincent J, Ascano M (2020). Small molecule inhibition of human cGAS reduces total cGAMP output and cytokine expression in cells. Sci Rep.

[69] Steinhagen F, Zillinger T, Peukert K, Fox M, Thudium M, Barchet W (2018). Suppressive oligodeoxynucleotides containing TTAGGG motifs inhibit cGAS activation in human monocytes. Eur J Immunol.

[70] Wang M, Sooreshjani MA, Mikek C, Opoku-Temeng C, Sintim HO (2018). Suramin potently inhibits cGAMP synthase, cGAS, in THP1 cells to modulate IFN-β levels. Future Med Chem.

[71] Dai J, Huang YJ, He X, Zhao M, Wang X, Liu ZS (2019). Acetylation blocks cGAS activity and inhibits self-DNA-induced autoimmunity. Cell.

[72] Harcourt JL, Offermann MK (2000). Interferon-alpha synergistically enhances induction of interleukin-6 by double stranded RNA in HeLa cells. Eur J Biochem.

[73] Henrickson M, Wang H (2017). Tocilizumab reverses cerebral vasculopathy in a patient with homozygous *SAMHD1* mutation. Clin Rheumatol.

